# Spatio‐temporal characteristics of the gastrointestinal resistome in a cow‐to‐calf model and its environmental dissemination in a dairy production system

**DOI:** 10.1002/imt2.70047

**Published:** 2025-05-14

**Authors:** Shuai Liu, Yimin Zhuang, Tianyu Chen, Duo Gao, Jianxin Xiao, Jinfeng Wang, Jinghui Li, Xinjie Zhao, Rong Peng, Wenli Guo, Jialin Wei, Mo Sha, Jingjun Wang, Jiaying Ma, Mei Ma, Mengmeng Li, Wei Wang, Ya‐Jing Wang, Shengli Li, Zhijun Cao

**Affiliations:** ^1^ State Key Laboratory of Animal Nutrition and Feeding, International Calf and Heifer Organization, College of Animal Science and Technology China Agricultural University Beijing China; ^2^ Animal Nutrition Institute Sichuan Agricultural University Chengdu China; ^3^ College of Food Science and Nutritional Engineering China Agricultural University Beijing China; ^4^ Department of Medicine, Section of Genetic Medicine University of Chicago Chicago Illinois USA; ^5^ Institute of Agricultural Sciences ETH Zurich Zurich Switzerland; ^6^ School of Biological Sciences University of Bristol Bristol UK

**Keywords:** antimicrobial resistance genes, dairy production system, gastrointestinal tract, microbiome transmission, multi‐omics analysis

## Abstract

Microbiome and resistome transmission from mother to child, as well as from animal to environment, has been widely discussed in recent years. Dairy cows mainly provide milk and meat. However, in the dairy production system, the characteristics and transmission trends of resistome assembly and the microbiome in the gastrointestinal tract (GIT) remain unclear. In this study, we sequenced the GIT (rumen fluid and feces) microbiome of dairy cow populations from two provinces in China (136 cows and 36 calves), determined the characteristics of their resistome profiles and the distribution of antibiotics resistance genes (ARGs) across bacteria and further tracked the temporal dynamics of the resistome in offspring during early life using multi‐omics technologies (16S ribosomal RNA [rRNA] sequencing, metagenome, and metatranscriptome). We characterized the GIT resistome in cows, distinguished by gut sites and regions. The abundance of ARGs in calves peaked within the first 3 days after birth, with *Enterobacteriaceae* as the dominant microbial host. As calves aged, resistome composition stabilized, and overall ARG abundance gradually decreased. Both diet and age influenced carbohydrate‐active enzymes and ARG profiles. Resistance profiles in ecological niches (meconium, colostrum, soil, and wastewater) were unique, resembling maternal sources. Mobile genetic elements (MGEs), mainly found in soil and wastewater, played an important role in mediating these interactions. Multidrug resistance consistently emerged as the most significant form of resistance at the both the metagenome and metatranscriptome levels. Several antibiotic classes showed higher proportions at the RNA level than at the DNA level, indicating that even low‐abundance gene groups can have a considerable influence through high expression. This study broadens our understanding of ARG dissemination in livestock production systems, providing a foundation for developing future preventive and control strategies.

## Highlights

- The gastrointestinal tract of dairy cows is a natural reservoir of resistomes and distinguished by gut sites and regions.
- In the early life, antibiotics resistance genes may acquire from maternal source, and diet and age are the primary regulatory factors of the resistome.
- Mobile genetic elements are an important medium between various ecological niches interactions which may occur most frequently from the soil and wastewater.
- Multidrug resistance consistently holds paramount significance as the most crucial form of drug resistance at the both metagenome and metatranscriptome level.

## Graphical abstract

This study revealed spatio‐temporal characteristics of gastrointestinal resistome in a cow‐to‐calf model and the pattern of their spread to the environment and offspring in the dairy production system. The gastrointestinal tract of dairy cows is a natural reservoir of resistomes and distinguished by gut sites and regions. The resistance profiles of different ecological niches (meconium, colostrum, soil, and water) were unique, and most of the features were shared with the maternal source. In the early life, antibiotics resistance genes may acquire from the maternal source, and diet and age are the primary regulatory factors of the resistome. Mobile genetic elements are an important medium between various ecological niches interactions which may occur most frequently from the soil and waste water.

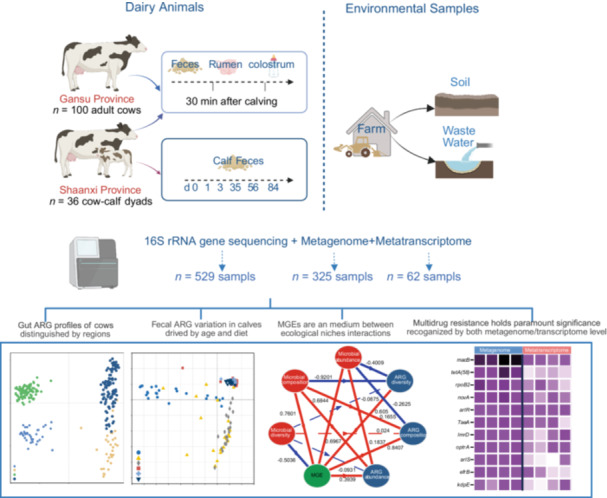

## INTRODUCTION

Microbial drug resistance is a global public health concern [1]. The widespread of antibiotics resistance genes (ARGs) in the environment and biosphere poses a serious threat to food safety and health [2], with animal husbandry being a primary source [3, 4]. Intensive dairy production with cows as the main farm animals often involves the use of antibiotics in the feeding process for improved productivity and disease resistance, contributing higher economic benefits to the pasture. However, this invariably leads to the prevalence of ARGs in the gastrointestinal tract (GIT) of dairy cows, potentially contaminating the environment (water and soil [ES], etc.) through fecal excretion or indirectly transmitted to humans through the food chain [5, 6]. Therefore, considering the importance of the GIT microbiome in dairy cow production system and its challenges as an ARG reservoir, it is crucial to understand the spatiotemporal characteristics of the resistome within dairy populations and the transmission patterns during the breeding process to formulate corresponding prevention and control strategies to reduce the associated risks and promote sustainable animal husbandry development.

Research has shown that the GIT of ruminants, notably the rumen, functions as a natural reservoir for ARGs [7, 8]. This environment hosts a microbial community that is not only more diverse but also uniquely adapted to high‐fiber diets compared with those of monogastric animals. This rich biodiversity, however, heightens the risk of ARG dissemination, and we need to reduce the use of antibiotics in animal feeding to control ARG spreading [9]. At present, ARG transmission primarily occurred through two mechanisms: horizontal gene transfer (HGT) and vertical transfer. HGT, aided by mobile genetic elements (MGEs), enables the exchange of ARGs among microbial populations across diverse ecological niches [10, 11]. Vertical transmission, on the other hand, refers to the transmission from mother to offspring [12]. Reportedly, the first transmission of the gut microbiome occurs during neonatal birth and has lifelong consequences [13, 14]. One study proved probiotic supplementation during pregnancy could improve the gut microbiome of sows and regulate the early‐life fecal microbiome development and maturation of piglets [15]. Similarly, ARGs carried by bacteria were also inherited by offspring during this process [16]. What deserves more attention is that the animal gut microbiome and resistome can alter the microbial ecology of the surrounding ES, water sources, and workers [17, 18, 19]. Although there have been some studies that investigated the spread and development of resistome in dairy cows, these studies tend to focus on a particular aspect (such as environmental [20] or offspring transmission [16]) and lack a comprehensive discussion of the interactions between these directions and the overall model. Addressing this issue is crucial to understand how to efficiently and accurately mitigate the spread of ARGs originating from the GIT of dairy cows.

In this study, using multi‐omics technology (16S ribosomal RNA [rRNA] sequencing, metagenome, and Metatranscriptome), we aim to sequence the (rumen and fecal content) microbiome of dairy cows from two provinces in China and determine the characteristics of their resistome profiles and ARG distributions across bacteria. We also undertake a thorough comparison of the microbial and resistome diversity and abundance across various niches, encompassing ES, water, neonatal meconium (ME), and secreted colostrum (CL). Furthermore, we seek to explore the associations between these niche microbiomes and maternal sources, focusing specifically on the levels of ARGs and MGEs. Finally, we will track the dynamics and proliferation patterns of gut resistome and function in the early life of these cows' offspring from birth to weaning.

## RESULTS

### The distribution of resistome along the GIT of dairy cows in different regions

**Figure 1:**
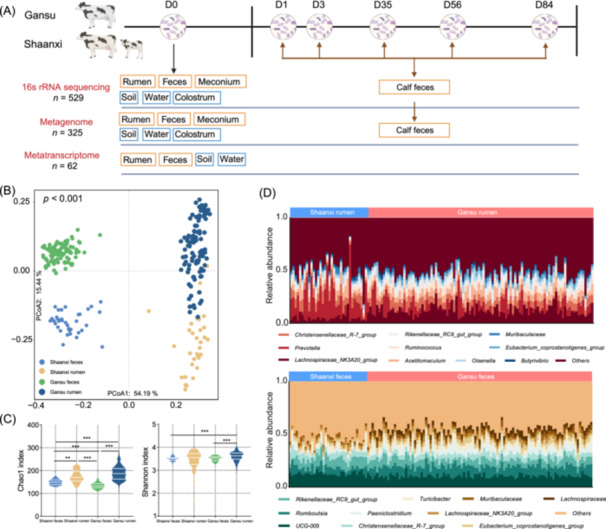
The rumen and hindgut microbiota of cows in Gansu and Shaanxi province. (A) Experimental design and sampling. D0–D84 represented that each cow and related environmental samples are sampled at their corresponding age points. These newborn calves were fed with colostrum from their dam within half an hour after birth and then transferred to the clean and tidy separate calf hutches. Calves were fed milk from the age of 1–3 weeks, fed milk replacer (Table S2) from the age of 3–8 weeks, and weaned at 56 days of age. After weaning, the calves were maintained in calf hutches until the end of the experiment at 84 days of age. The calves had unrestricted access to water and starters (Table S3) from birth to 84 days of age. (B) Principal coordinates analysis (PCoA) based on the Bray–Curtis distance of microbiota in the rumen and feces of cows from Gansu and Shaanxi. (C) The α diversity of microbiota in the rumen and feces of cows from Gansu and Shaanxi. The histogram indicated the expression levels of these genes at different ages (*p < 0.05, **p < 0.01, ***p < 0.001). (D) The microbial composition of rumen and feces at the genus level in cows from Gansu and Shaanxi.

Describing taxonomic changes is the first step in exploring the GIT microbiome of animals. In this study, 134 rumen contents and 136 fecal samples were collected from 136 parturient dairy cows in the Gansu (*n* = 100) and Shaanxi (*n* = 36) provinces (Figure 1A). To assess the microbial profiles of the samples, 16S rRNA gene sequencing was performed. The results revealed a distinct difference in the geography and gut region of the cow microbiome (Figure 1B). The rumen microbiome significantly differed from that of the feces (*p* < 0.001) and across different geographical locations (*p* < 0.001). Regarding alpha diversity, rumen samples showed higher Chao1 and Shannon indices than feces (Figure 1C; *p* < 0.05). *Lachnospiraceae_NK3A20_group*, *Prevotella*, and *Christensenellaceae_R‐7_group* were the dominant genera in rumen samples, whereas *UCG‐005*, *Romboutsia*, and *Rikenellaceae_RC9_gut_group* were abundant in feces (Figure 1D).

**Figure 2:**
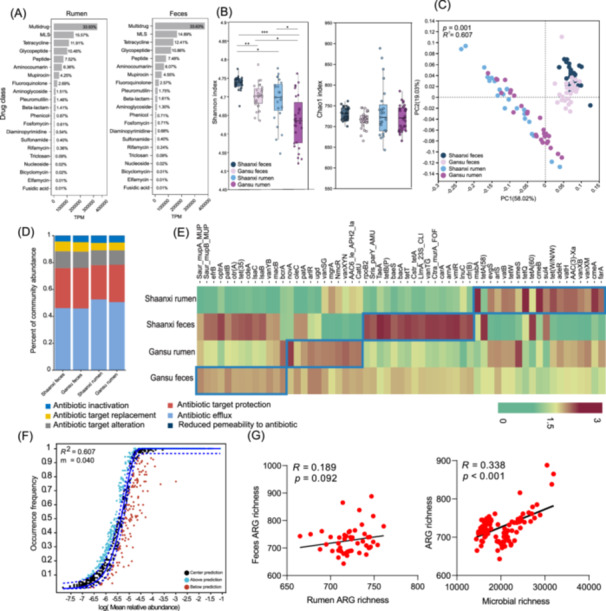
The gut resistome profile of cows in Gansu and Shaanxi. (A) The total abundance of antibiotics resistance genes (ARG) resistance to 21 drug classes in the rumen and feces. (B) The α diversity of resistome in the rumen and feces of cows from Gansu and Shaanxi. The boxplot indicated the expression levels of these genes at different ages. (*p < 0.05, **p < 0.01, ***p < 0.001). (C) Principal coordinates analysis (PCoA) based on the Bray–Curtis distance of resistome in the rumen and feces of cows from Gansu and Shaanxi. (D) The composition of resistance mechanisms in the rumen and feces. (E) The identification of signature ARGs in the four groups. The abundance values were log‐transformed (log [transcripts per million (TPM) + 1, 10]) for better visualization. (F) Fit of neutral model determined the contribution of deterministic and stochastic processes on ARGs. (G) The correlation analysis (feces ARG richness vs. rumen ARG richness; ARG richness vs. microbial richness).

Next, we characterized ARGs in the rumen and feces of two dairy cows in Gansu and Shaanxi provinces. A total of 1194 ARGs were identified, conferring resistance to 21 different drug classes. Notably, ARGs associated with multidrug resistance were the most prevalent in rumen and feces, followed by resistance to macrolide‐lincosamide‐streptogramin B (MLS), tetracycline, glycopeptide, and peptide (*p* < 0.05*;* Figure 2A). For resistome diversity, the fecal resistome had a higher Shannon index than the rumen, and the rumen resistome in Gansu was the lowest among the four groups (*p* < 0.05; Figure 2B). No significant difference in the Chao1 index was observed between the groups. There were distinct resistome structures regarding β‐diversity based on the GIT at the region of collection (*R* ^*2*^ = 0.607, *p* < 0.05; Figure 2C). Analysis of resistance mechanisms revealed a similar composition across the four groups, with antibiotic efflux being the highest percentage, followed by antibiotic target alteration, protection, replacement, and antibiotic inactivation (Figure 2D). The signature ARGs in each group were identified using linear discriminant analysis (LDA) effect size (LEfSe; LDA > 2; *p* < 0.05), as shown in the heatmap (Figure 2E). *Saur_mupA_MUP*, *efrB*, *optrA*, and *tet (35)* were the signatures in the Gansu feces group, and *novA*, *patA*, *arlR*, and *mgrA* were the dominant signatures in the Gansu rumen group. In the Shaanxi fecal group, *Sris_parY_AMU*, *TaeA*, *tetB(P)*, *baeS*, and *tetT* were the signature ARGs, while he*msbA*, *tetQ*, *cmeB*, and *cmeA* signatures were abundant in the Shaanxi rumen group. The neutral community model (NCM) is specifically tailored to discern the influence of random processes on the aggregation patterns within microbiome [21, 22]. It accomplishes this by scrutinizing the interplay between the occurrence frequency of taxa and their relative abundance. In this study, this model was applied to quantify the hypothesis that stochastic events including diffusion and drift, are pivotal in sculpting the structural landscape of microbial resistome. As shown in Figure 2F, Figure S1, the NCM successfully estimated most of the relationship between the occurrence frequency of ARGs and their abundance, showing a high interpretation rate in either the four groups or all sample sets (*R* ^*2*^ = 0.989; *R* ^*2*^ = 0.952, *R* ^*2*^ = 0.959, *R* ^*2*^ = 0.895, and *R* ^*2*^ = 0.927), suggesting that the random process was critical for the formation of resistome assembly in different GIT and geographical locations. Further, we calculated the correlations between rumen and fecal ARG using Spearman analysis. Although no significant correlation was observed between the two groups (Figure S2A), ARG richness between the two tracts showed a trend toward a significant correlation (*R* = 0.189; *p* = 0.092; Figure 2G). The significant correlations between microbial and ARG richness were confirmed in Gansu, Shaanxi, and all samples (*p* = 0.002, *p* = 0.047, and *p* < 0.001, respectively; Figure S2B,C, Figure 2G).

**Figure 3:**
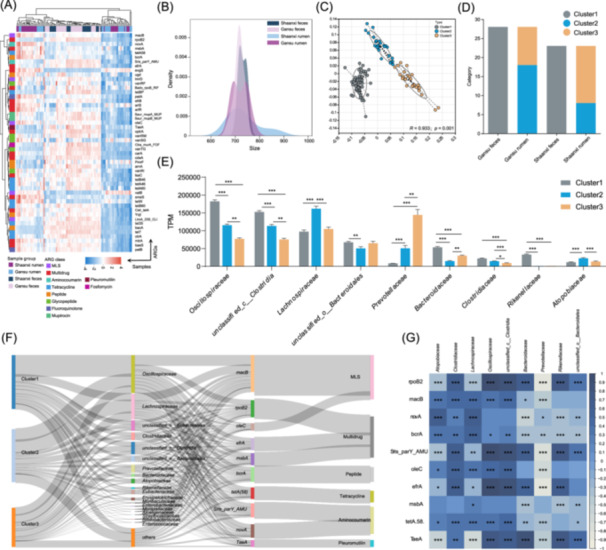
Characteristics of three resistome clusters associated with feces and rumen. (A) The heatmap depicting sample clustering based on the first 50 abundance values of antibiotics resistance genes (ARG) abundances. The ARGs were standardized by *z*‐score, and *z* = (*x*–*µ*)/σ. The transcripts per million (TPM) of ARGs (*x*) in each sample is subtracted from the mean (µ) and then divided by the standard deviation (σ). (B) Density plot of ARG richness in the cow cohort. (C) ARGs were clustered with partitioning around medoids clustering based on Euclidean distance. (D) The proportion of different clustering samples in the four groups. (E) The significant difference of the top 10 microbial families among the four clusters. The bar graph indicated the expression levels of these genes at different ages (*p < 0.05, **p < 0.01, ***p < 0.001). (F) Sankey diagram connecting the predicted bacterial hosts (2nd column) from the three clusters (1st column) to the top 10 ARGs (3rd column) in the six drug classes (4th column). (G) Spearman correlation analysis between ARGs and bacteria (*p < 0.05, **p < 0.01, ***p < 0.001).

Based on the top 50 ARG abundance patterns, the GIT samples were divided into three nonoverlapping groups (Figure 3A), and the distribution of ARG richness in the Gansu rumen group was bimodal (Figure 3B). Similarly, according to the Calinski Harabasz Index of partitioning around medoids, ARG profiling of samples based on abundance demonstrated an optimal number of three clusters (*k* = 3), demonstrating the robustness in the cow resistome (Figure S3). All fecal samples were grouped into one cluster (Cluster1), and the rumen samples from Gansu and Shaanxi were grouped into two clusters (Cluster2 and Cluster3; Figure 3C,D). The α diversity revealed no difference in the Chao1 index among the clusters, with Shannon index values in Cluster1 and Cluster3 higher than in Cluster2 (Figure S4). We compared the abundance of the main bacteria at the family level among the three clusters and found that *Oscillospiraceae*, *Bacteroidaceae*, *Clostridiaceae*, and *Rikenellaceae* were the dominant families in Cluster1 (*p* < 0.05), whereas *Lachnospiraceae* and *Atopobiaceae* were enriched in Cluster2 (*p* < 0.05). *Prevotellaceae* predominated in Cluster3 (*p* < 0.05; Figure 3E). Moreover, we assessed the contribution of these bacteria as hosts for the top 10 ARGs in the resistome (Figure 3F). *Oscillospiraceae, Lachnospiraceae*, *Clostridiaceae*, and *Prevotellaceae* were the main resistome‐related families, and the contributions of microbial hosts varied greatly among the different cluster groups. For instance, *Prevotellaceae*, as a host, showed a higher contribution to ARGs in Cluster 3 than in Clusters 1 and 2. Correlation analysis demonstrated a significant association between potential bacterial hosts and major ARGs (*p* < 0.05; Figure 3G).

### Temporal dynamics of the fecal resistome and carbohydrate function in offspring

**Figure 4:**
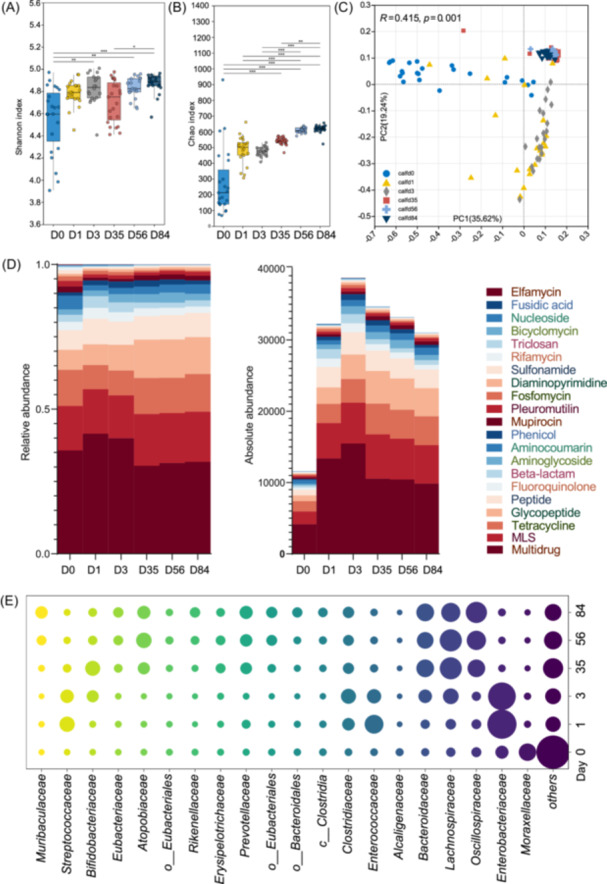
The temporal dynamics of fecal resistome in calves. (A and B) The Chao1 index (A) and Shannon index (B) of fecal antibiotics resistance genes (ARG) in calves on Day 0, 1, 3, 35, 56, and 84. The histogram indicates the expression levels of these genes at different ages. (*p < 0.05, **p < 0.01, ***p < 0.001). (C) Principal coordinates analysis (PCoA) based on the Bray–Curtis distance of resistome in the feces of calves. (D) The relative abundance of fecal ARGs at different ages and the absolute abundance (Transcripts Per Million [TPM]) of fecal ARGs at different ages. (E) The predicted bacterial hosts of fecal ARGs at different ages.

To further understand the temporal evolution of the fecal resistome in dairy offspring during the early life, we tracked the presence of ARGs in neonatal calves of Shaanxi cows, as these calves were remained to raising in the farm of Shaanxi. Regarding taxa annotation, *Enterobacteriaceae* was the dominant family in the gut of calves on Days 1 and 3, followed by *Enterococcaceae* and *Streptococcaceae*, whereas these three bacteria disappeared after 35 days. The abundance of *Prevotellaceae*, *Oscillospiraceae*, *Moraxellaceae*, and *Clostridiaceae* increased with ages from Day 35 to 56. Conversely, *Bifidobacteriaceae* and *Coriobacteriaceae* decreased with age (Figure S5). Next, we examined the fecal resistome of calves with age. During the early life of calves, 1146 ARGs were detected in the fecal microbiome. The α‐diversity of resistome increased with age. The Shannon index on Days 56–84 was significantly higher than that on Day 0 (*p* < 0.05; Figure 4A). The Chao1 indices on Days 35, 56, and 84 were higher than those on Days 0, 1, and 3 (*p* < 0.05; Figure 4B). Consistent with the gradual variation in the microbial community, the resistome structure of the calves changed with increasing age (*p* = 0.001; Figure 4C). The samples of Days 0, 1, and 3 showed a large degree of dispersion, whereas from Days 35 to 84, the samples were tightly clustered, suggesting that an increase in age decreased the difference in the resistome among cows in the same population. In terms of the antibiotic class, the composition of the resistance was relatively stable at each age. Multi‐drug was the most prevalent antibiotic resistance across all ages, followed by MLS, Tetracycline, and Peptide (Figure 4D). Notably, the ME microbiome harbored the lowest abundance of ARGs, which peaked at Day 3. Thereafter, the total ARG abundance gradually decreased with age, which was mainly attributed to the decline in the abundance of multidrug‐resistant bacteria (Figure 4D). Furthermore, we tracked the age‐related changes in the main bacteria harboring ARGs at the family level in the feces of calves (Figure 4E). *Enterobacteriaceae*, *Streptococcaceae*, and *Enterococcaceae* were the most dominant hosts of ARGs on Days 1 and 3. *Bifidobacteriaceae* was characterized as the main ARG carriers on Day 35, while *Bacteroidaceae*, *Lachnospiraceae*, *Oscillospiraceae*, and *Prevotellaceae* mainly carried ARGs from Days 35 to 84 in increasing abundance. Next, random forest mode was used to identify time‐associated signature ARGs. As expected, these signatures conferred resistance to MLS, multidrug, tetracycline, glycopeptide, beta‐lactam, diaminopyrimidine, aminoglycoside, and rifamycin (Figure S6A), which showed distinct synchronization with the physiological age of the calves (*R* ^*2*^ = 0.980; Figure S6B), with CfxA2 being the most important variable that differed with age, followed by *ceoB*, *tet(C)*, *mel*, and *tet(44)*. Permutational multivariate analysis of variance indicated that age and diet were significantly correlated with the resistome profile, explaining 40.0% of the total variance (*p* < 0.05; Figure S6C). Similar to the cow resistome, the NCM proved that randomness was an important driving force shaping the gut resistome of calves of different ages (Figure S7).

**Figure 5:**
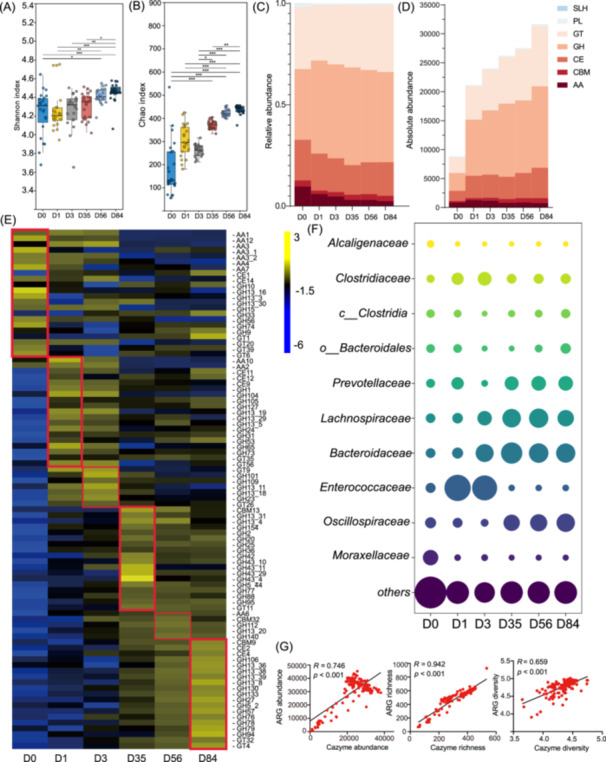
Functional capacity of gut microbiome in the calves. (A and B) The Chao1 index (A) and Shannon index (B) of fecal carbohydrate‐active enzymes (CAZyme) in calves on Days 0, 1, 3, 35, 56, and 84. The histogram indicates the expression levels of these genes at different ages (*p < 0.05, **p < 0.01, ***p < 0.001). (C) The relative abundance of fecal CAZymes at different ages. (D) The absolute abundance (transcripts per million [TPM]) of fecal CAZymes at different ages. AA, auxiliary activities; CBM, carbohydrate‐binding modules; CE, carbohydrate esterases; GH, glycoside hydrolases; GT, glycosyl transferases; PL, polysaccharide lyases; SLH, cellulosome modules. (E) The identification of signature CAZymes in the four groups. The abundance values were log‐transformed (log [TPM + 1, 10]) for better visualization. (F) The predicted bacterial hosts of fecal CAZymes at different ages. (G) The correlation analysis (ARG abundance vs. CAZyme abundance; ARG richness vs. CAZyme richness; ARG diversity vs. CAZyme diversity).

In terms of metabolic function, we investigated the profiles of carbohydrate‐active enzymes (CAZymes) in calves at different time points. The richness and diversity of the CAZymes increased with age (*p* < 0.05; Figure 5A,B). Glycoside hydrolases (GH) were the most abundant CAZyme class across the ages, followed by glycosyl transferases (GT), carbohydrate esterases (CE), auxiliary activities (AA), and carbohydrate‐binding modules (Figure 5C). The total absolute abundance of CAZymes was lowest on Day 1 and increased until Day 84 (Figure 5D). We found that the differential abundance of CAZymes changed with age (LDA > 2; *p* < 0.05; Figure 5E). On Day 0, the most differentially expressed CAZymes belonged to AAs, GHs, and GTs, including AA1, AA3, AA4, and GH10. GH13_16, GH13_3, GT20 GT39, and GT6. GH104, GH24, GH65, GH101, GH109, and GH26 were the dominant CAZymes on Days 1 and 3. The GH43 members were enriched on Day 35, while GH130, GH133, GH27, GH57, and GH95 were highly abundant from Days 56 to 84. We predicted the bacterial origin of CAZyme. Like the microbial hosts of ARGs, *Moraxellaceae* was the main host on Day 1, and *Enterococcaceae* contributed to the CAZymes on Days 1 and 3. The contributions of *Lachnospiraceae*, *Bacteroidaceae*, and *Prevotellaceae* were higher on Days 35, 56, and 84 (Figure 5F). CAZymes showed a close connection (*p* < 0.001) with ARGs in terms of abundance (*R* = 0.746), richness (*R* = 0.942), and diversity (*R* = 0.659; Figure 5G).

### Microbial transmission from the dairy cow into the environment and offspring

**Figure 6:**
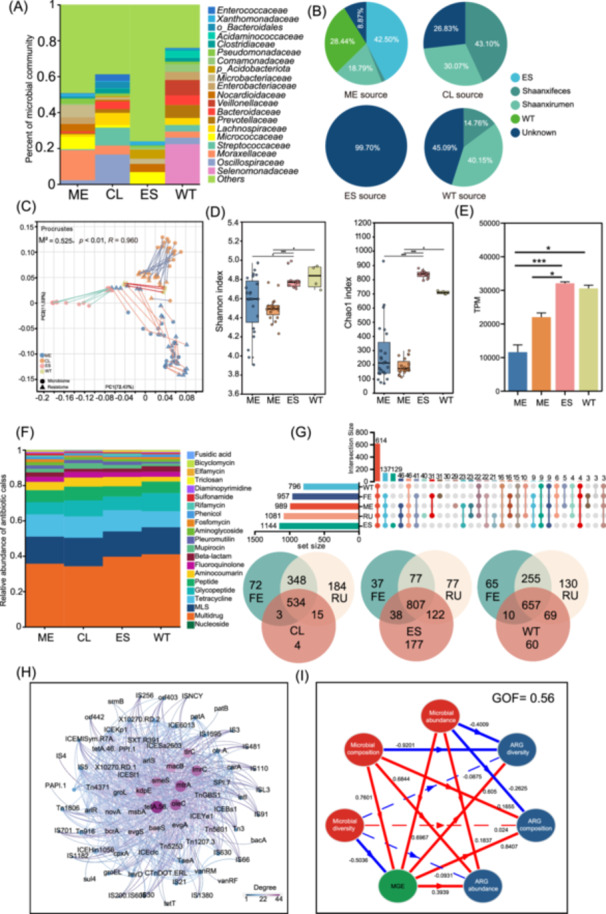
The resistome characteristics of meconium, colostrum, wastewater, and soil. (A) The microbial composition of four groups at the genus level. ME = Meconium; CL = Colostrum; ES = Soil; WT = Wastewater. (B) The SourceTracker analysis of microbiome in the four groups. (C) Procrustes analysis of the association between the microbial composition and ARG profile at the four groups. (D) The α diversity of microbiota in the four groups. (E) The abundance of antibiotics resistance genes (ARGs) in the four groups. The histogram indicated the expression levels of these genes at different ages (*p < 0.05, **p < 0.01, ***p < 0.001). (F) The composition of drug class of ARGs in the four groups. (G) Wayne diagram and upset plot showing the intersection of feces, rumen, and meconium/colostrum/soil/wastewater. (H) The Co‐occurrence network between ARGs and mobile genetic elements (MGE). (I) Partial Least Squares Path Modeling (PLS‐PM) to investigate direct and indirect effects of the microbiome, MGEs, and ARGs. The solid line indicated a significant causal relationship between the two factors, while the dashed line indicated no significant relationship between the two factors.

Regarding the microbial diversity of 16S sequencing, the ES group showed the highest values of Chao1 and Shannon indices. Compared with the samples from the CL and neonatal ME, the microbial richness of the environmental samples (ES) and wastewater (WT) were significantly higher (*p* < 0.05; Figure S8A). The microbial communities in these four niches showed distinct distances (*p* < 0.05; Figure S8B). To evaluate whether the GIT microbiota and resistome of pregnant dairy cows contributed to the surrounding environment (ES and WT) mammary secretions (CL and ME), we performed a metagenomic analysis of these samples. The microbial composition differed remarkably among the four groups. *Psychrobacter* and *Nocardioides* were the dominant genera in the ME, while *Oscillospiraceae* and *Lactococcus* were enriched in the CL. The WT contains a complex bacterial composition, with the main genera being *Selenomonas* and *Prevotella*. *Acidobacteriota* was the most abundant genus in ES, followed by *Nocardioides* and *Arthrobacter* (Figure 6A). SourceTracker algorithm was used to detect the maternal origin of the microbiome in these groups [23, 24] (Figure 6B). ES (42.50%) and WT (28.44%) were the main bacterial sources for the ME microbiome, whereas the rumen, as the maternal source, accounted for only 18.79% of the ME. For CL, the maternal source (rumen and feces) was the main microbial source, accounting for >70%. Similarly, WT also contained a significant proportion of maternal microbes, with a contribution of >50%. The maternal microbiome contributed little to the construction of ES microbial communities (<1%), suggesting that its unique microbial structure distinguished it from other sites. Moreover, like the microbiota profile, the resistome showed distinct differences across groups. Procrustes analysis confirmed that the microbial community was strongly correlated with the antibiotic resistome (*p* < 0.01) (Figure 6C). The Chao1 and Shannon indices were higher in ES and WT than in ME and CL (*p* < 0.05) (Figure 6D). Notably, the absolute abundance of ARGs in the ES was the highest among the four groups and was significantly higher than that in the ME and CL groups (*p* < 0.05; Figure 6E). Multidrug was the common resistance across groups, followed by resistance to MLS, Tetracycline, Glycopeptide, Peptide, Aminocoumarin, and Fluoroquinolone (Figure 6F). The Venn diagram shows the shared and unique features of the different niche resistomes (Figure 6G). Most ARGs of ME could be shared with rumen and feces. A total of 23 and five ARGs in ME were shared with ES and WT, respectively, whereas only nine ARGs were shared among the ME, ES, and WT. Almost all ARGs in the CL were shared by maternal sources. For the environmental samples, although ES and WT shared more ARGs with the rumen and feces than ME and CL, a higher number of unique ARGs were observed at these two sites. Specifically, the ES samples contained 177 ARGs that were exclusively missing from the maternal sources.

To explore the diffusion potential of the resistome in these different niches, we identified the MGE and constructed a co‐occurrence network of ARGs and MGEs. The network displayed frequent connections between MGEs and ARGs (Figure 6H). Moreover, partial least squares path modeling was used to detect potential correlations between the microbiome, resistome, and MGEs (goodness‐of‐fit = 0.56; Figure 6I). The results indicated that the microbial composition and abundance had a direct positive effect on the abundance of MGE and ARG, whereas the opposite trend was observed for ARG diversity. The MGE abundance was also positively correlated with ARG abundance, composition, and diversity (*p* < 0.05). We compared the abundance of ARGs and MGEs, which were hub nodes in the co‐occurrence network. Consistent with the total abundance, most ARGs, including *macB*, *oleC*, *tetA(58)*, *smeS*, *tlrC*, and *ImrC* were the most abundant in ES, whereas *kdpE* showed the highest abundance in WT. All ARGs in ME maintained the lowest abundance compared with the other three groups. Hub MGEs consistently showed the highest abundance in WT, followed by ES (Figure S9).

Next, the gut samples of six cows and their offsprings and eight related environment samples (four ES samples and four wastewater samples) were randomly selected to explore the potential horizontal transfer of ARGs by metagenomic binning analysis. As the result, we identified a total of 264 metagenome‐assembled genomes (MAGs) of medium and high quality (more than 50% completeness and less than 10% contamination) and further annotated them into MGE and CARD databases. According to the gene potential mobile analysis maps, two representative ARGs (*macB* and *tetA(58)*) were shared among different species‐level MAGs in the cow, calf, ES, and WT and all showed the close proximity to MGE in the same contig sequence, which could suggest the HGT ability of ARGs in the different niches of this study (Figure S10).

Moreover, on the basis of emphasizing the prevalence of ARG transmission in these niches, we further fully assessed the potential ecological risks of the spread of these components according to the method of “MetaCompare” (Figure S11) [25]. As the result, WT samples showed the highest risk score among the groups, followed by ES, cow feces, and rumen (*p* < 0.05). The risks of CL and ME were the lowest (*p* < 0.05). Of note, after the CL administration, the risk of calf feces displayed an upward trend.

### The comparison of DNA‐based and RNA‐based resistome profiling in the different sample types

**Figure 7:**
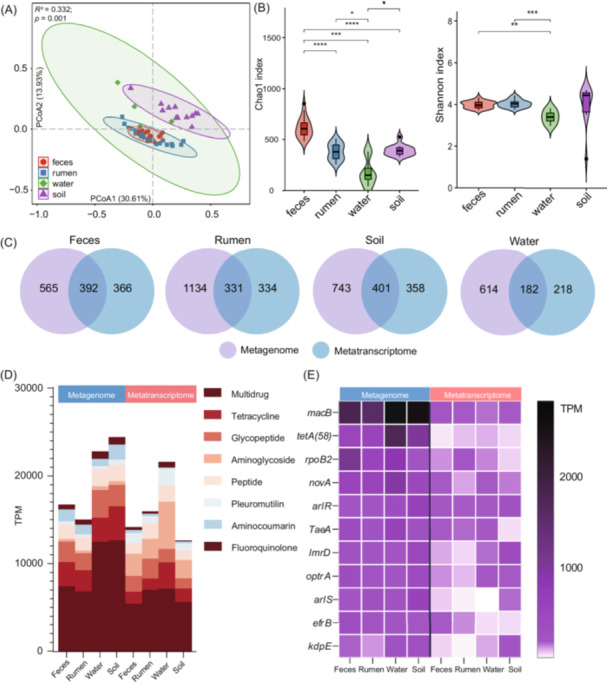
The comparison of antibiotics resistance genes (ARG) at DNA‐based and RNA‐based level. (A) Principal coordinates analysis (PCoA) based on the Bray–Curtis distance of ARG transcriptional abundance in the groups of feces, rumen, soil, and water. (B) The Chao1 index and Shannon index of four groups at the metatranscriptome level (*p < 0.05, **p < 0.01, ***p < 0.001, ****p < 0.0001). (C) The Venn diagram of ARG identification between the metagenome and metatranscriptome level. (D) The comparison of antibiotic class of ARGs between metagenome and metatranscriptome. (E) The comparison of ARG abundance between metagenome and metatranscriptome.

To determine the activity of ARGs throughout the dairy production system, metatranscriptomic (RNA‐based) sequencing and analyses were conducted to evaluate the expression levels of these ARGs. Consistent with the profile of metagenomic sequencing, the structure and diversity of resistome among the feces, rumen, ES, and WT showed the significant difference (*p* < 0.05) (Figure 7A,B). Specifically, the maximal distance disparity was observed between GIT samples of cows and environmental samples, and the WT samples displayed the lowest richness (Chao1 index) and diversity (Shannon index). We further detected the ARGs that were either shared or unique between the two omics data sets within each respective niche (Figure 7C). The metagenome identified a greater number of ARGs compared with the metatranscriptome. At the metatranscriptome level, only around half of the transcripts could be annotated according to the metagenome and the remaining genes were only detected at either the DNA or RNA level, but not both. In terms of antibiotic class, multidrug resistance consistently hold paramount significance as the most crucial form of drug resistance at both metagenome and metatranscriptome level, while some categories including Aminoglycoside, Aminocoumarin, and Pleuromutilin showed the distinct difference in abundance between the two omics (Figure 7D). Finally, we observed either consistent or distinct ARG signatures of the four groups between metagenome and metatranscriptome. For instance, *macB* and *tetA (58)* were the most abundant in the ES and WT of metagenome, which differed from the result of metatranscriptome. However, the trends of *kdpE*, *novA*, and *arlR* were consistent in the metagenome and metatranscriptome (Figure 7E).

## DISCUSSION

Dairy cattle, a prominent livestock species globally, possess a specialized GIT and a distinct microbiome, and this unique combination allows them to efficiently utilize high‐fiber diets, which are indigestible for monogastric animals, and convert these forages into high‐quality animal protein [26, 27]. However, their guts might be a reservoir of ARGs carried by the microbiome, posing a huge threat to the environment and human beings as well as to sustainable development [8]. Therefore, understanding the spatiotemporal characteristics of the GIT resistome of dairy cows and evaluating its tendency to spread outward in the production system is crucial to formulating coping strategies that reduce contamination with resistance genes.

In this study, we determined the significant difference between the taxonomy of rumen and hindgut microbiota in dairy cows, as confirmed in previous studies. *Prevotella*, *Ruminococcus*, and *Butyrivibrio*, as fiber decomposers, are enriched in the rumen in response to a high‐cellulose diet [28, 29] and are further converted into volatile fatty acids and high‐quality microbial proteins for host maintenance and production [30, 31]. The rumen microbiota exhibited higher richness than the feces. This might be attributed to the higher redundancy of the rumen microbiome, which could ensure the high stability of microbial functions with anti‐disturbance abilities [32, 33]. For the resistome, multidrug was the most dominant antibiotic resistance class in rumen and feces. Reportedly, bacteria with multidrug resistance show higher tolerance to traditional antibiotics [34], which poses a challenge to the antibiotic treatment of dairy cows. Tetracycline and MLS were identified as the main resistance classes. Studies have confirmed their prevalence in the gut of humans and livestock [35], although some individuals never received the necessary antibiotic treatment [36], suggesting that GIT might be the natural ARG reservoir [37], which could provide some reference for other populations. The potential causes of the generation of ARGs in dairy cattle are a complex issue with multiple potential contributing factors, including antibiotic use, HGT, agricultural practices, GIT microbiome, environmental factors, and so on. The high percentage of these ARGs may not only be caused by antibiotic selective pressure but also by horizontal transmission due to high‐density feeding patterns in China's large‐scaled farm. Moreover, we observed resistome specificity between the rumen and feces, which may depend on the distinct microbial structures of these two gut sites. As an ARG host, the characteristics of the microbiome are typically closely associated with its resistome. In this study, there was a significant positive correlation between bacterial diversity and ARGs. Notably, we detected a significant association of ARG richness between the rumen and feces, suggesting the fluidity of ARGs among the different GITs. In addition to GIT specificity, the resistance profiles of the cows were also influenced by region, as indicated by the geographical effect in both cows and humans [38]. Dietary regimes can effectively regulate the composition and abundance of resistomes in animals [39, 40]. In this study, variations in diet ingredients and nutrient composition between Shaanxi and Gansu cows might be partly due to local dietary strategies. Additionally, the random process was an important driving force for the assembly of the GIT resistome in dairy cows, according to the NCM results. Cluster analysis revealed the subgroups within the rumen resistome in dairy cows, and the composition of the microbial hosts of the same ARGs in different clusters varied greatly. This emphasized that the resistance elements were not unidirectional but could flow in other taxa, thereby aggravating their diffusion and proliferation in the microbiome [41]. Additionally, cluster analysis was conducted to classify the rumen resistome of cows into two subgroups that were not influenced by the region. The differences in rumen microbial structure in different clusters may be the main reason for differentiating the related resistomes. As expected, the major bacteria in the clusters showed significant differences in abundance. The microbial contribution of the resistome and the correlations between bacteria and ARGs revealed that the main microbial hosts of ARGs were usually the dominant bacteria with high abundance in each cluster. These results emphasize that the flow and proliferation of the microbiome are important reasons for ARG spread in the gut of cows.

Next, we detected the prevalence of fecal ARGs in the offspring of Shaanxi dairy cows on Days 0, 1, 3, 35, 56, and 84. In terms of the microbial profile, richness, diversity, and composition all showed distinct differences across these time points. The Chao1 and Shannon indices of the calf ME (Day 0) were the highest, and the structure showed a significant distance compared with the other groups. The richness and diversity of ME significantly decreased within 24 h after these calves were born, which might be related to the specificity of the hindgut environment in calves. Although the hindgut of newborn calves is frequently invaded by maternal and environmental microbiota after birth, the specific pH, temperature, and gas in the hindgut quickly remove most of the microbes that are not suitable for colonization in this niche in a short time [42]. Moreover, the abundance of *Escherichia Shigella* on Days 1 and 3 decreased sharply from Days 35 to 84. In the previous studies, the calves had a high incidence of diarrhea in the first week after birth, which might be directly related to the high abundance of *Escherichia Shigella* in the hindgut at this stage. However, with the improvement in hindgut physiology and the formation of a strict anaerobic environment, *Escherichia Shigella* no longer occupies a favorable niche, and its proliferation is inhibited by other bacterial competitors [32, 43]. Conversely, owing to the intake of more solid feed with age, the abundance of several nutrient‐decomposing bacteria increases significantly to cope with high concentrations of nutrients in calf diets [44, 45]. Further, weaning calves at 70 days led to their hindgut microbiota resembling that of adult cows. The dominant bacteria were *Rikenellaceae_RC9_gut_group* and *UCG‐005*, which are consistent with previous studies [46, 47]. In terms of the fecal resistome, distinct differences in diversity and structure were observed among the groups. Notably, the absolute abundance of ARGs peaked on Days 1 and 3; considering that *Enterobacteriaceae* was the main microbial host of ARGs at these two‐time points, the high abundance of the resistome might be closely related to the high abundance of *Escherichia Shigella*. It is noteworthy that the abundance of ARGs in the gut of newborn calves peaked within the first 1–3 days after birth, coinciding precisely with the period immediately following the feeding of CL. This temporal alignment prompted us to hypothesize that microbiota carrying a substantial number of ARGs in CL might facilitate vertical transmission from the dam to the offspring through CL administration. Consequently, this transmission mechanism rapidly elevated the abundance of ARGs in the gut of calves. Thus, CL emerged as a crucial and timely contributor to the establishment of the gut resistome of newborn calves within a short timeframe. Although these calves had never been treated with antibiotics in the initial first week, our study proved that the hindgut might be a natural repository of ARGs [48, 49]. We observed significant temporal characteristics of hindgut ARGs in calves, and diet was the main driving factor, implying that manipulating the gut resistome of calves through dietary strategies may be a potential strategy for limiting ARG spread. For instance, feeding calves with milk replacer instead of breast milk could significantly reduce the diversity and richness of rumen resistome in calves [27]. Additionally, CAZyme profiles of the calf hindgut were influenced by the diet. As different diets change with age (transition from milk to starter), calves must constantly adjust the structure of their gut microbiota and microbiome function to metabolize different types of carbohydrates [8]. Therefore, changes in the resistome may be highly associated with the functional categories of microbial metabolism. Our study revealed a strong correlation and overlap between functional genes and ARGs regarding diversity, richness, and potential microbial hosts.

By comparing ARGs at different sites, we identified unique and shared features of the resistome in these niches. Compared with the biological samples (ME and CL), the samples of environmental niches (WT and ES) showed a higher richness and abundance of the resistome, which might be attributed to the fact that ME and CL were samples of low biomass [50, 51], resulting in fewer microbes carrying ARGs. More importantly, some studies have proved the widespread existence of ARGs in these environments may weaken some ecological functions, such as the maintenance of ES fertility [52] and the biodegradation of pollutants [53], and the loss of these functions will have a long‐term effect on the environment and human welfare. Moreover, traceability analysis proved that the contribution of the maternal microbiome to the CL was the most obvious, which accounted for >60%, emphasizing the frequent invasion of the maternal microbiome into the milk secreted by the mammary glands of cows. Considering the significant role of milk as an essential component in the diet of humans and other animals, the accumulation of ARGs carried by microbiota in milk within the food chain might lead to enhanced drug resistance in organisms at higher trophic levels within ecosystems [54], which could exert profound impacts on the structure and function of the entire food web. Therefore, before the entry of raw milk into the market, conducting necessary sterilization and quality inspection procedures on raw milk serves as an essential basis. For the ME, although a vertical transmission effect was also detected in this study [55], the interference of the environment on its microbial community could not be ignored.

Of note, the horizontal transfer by MGEs might promote the diffusion of ARG in these niches. Therefore, we determined frequent positive correlations among microbes, ARGs, and MGEs according to the results of the structural equation and the co‐occurrence interaction network. The core nodes of ARGs and MGEs in the network were enriched in ES and WT, implying that they may be important origins for spreading ARGs, which is consistent with previous studies [56, 57]. Partial Least Squares Path Modeling (PLS‐PM) analysis identified bacterial communities as primary hosts for ARGs and MGEs. Niche alterations within these communities likely exert a significant influence on the dissemination and distribution of ARGs and MGEs. Furthermore, potential HGT events among different niches, as suggested by mobile ARG analyses, emphasize the critical role of MGEs in facilitating ARG spread. This understanding suggests that manipulating microbial community structure within specific niches through exogenous interventions, such as the introduction of specific bacterial strains [58], anti‐antibody products [59], or altering environmental conditions [60], could potentially offer novel strategies for mitigating the dissemination of ARGs and MGEs. These approaches could be explored as a strategy to manage or mitigate the spread of antibiotic resistance in ecological systems. Moreover, we conducted an assessment of the subsequent risk associated with the dissemination of these distinct niche resistome [25]. Compared with the GIT samples, environment resistome (water and ES) showed a higher ecological consequence, which was consistent with the co‐occurrence network. Previous studies have also demonstrated that contamination of water or flooring surfaces with animal feces can markedly elevate the diversity of gut resistome in the nearby individuals [61, 62]. Therefore, based on this compelling evidence, it is imperative to conduct timely and thorough environmental cleaning of dairy production systems. Such practices could potentially mitigate the adverse impacts of environmentally resistome on the surrounding ecology and safeguard the gut health of dairy farmers.

Finally, we investigated the expression level of the resistome by metatranscriptome sequencing and paid attention to the notable difference characteristics of ARGs at the DNA and RNA level, respectively, which might be attributed to that the metagenome did not fully capture all the genetic diversity present in the resistome or some ARGs might be regulated by transcriptome factors and thus were not expressed at the RNA level [63]. In addition, we observed several antibiotic classes showed a higher proportion at the RNA level than that at the DNA level. This finding indicated that even gene groups of low abundance can exert considerable influence via their high expression, underscoring the necessity of meticulously examining concurrent changes at both the DNA and RNA levels in future comparable investigations. This approach will enable a more thorough elucidation of the roles these genes play within their respective biological contexts.

Although an investigation was conducted comprehensively in the characteristics and transmission patterns of resistome in dairy cow production systems in this study, there is still a lack of further research to evaluate the effectiveness of targeted exogenous interventions in limiting the spread of ARGs based on these findings, which is worthy of our next exploration.

## CONCLUSION

This study comprehensively characterized the distribution and dissemination of ARGs within dairy production systems. The GIT of dairy cows serves as a natural reservoir for diverse resistomes, influenced by regional and site‐specific factors. Maternal origins and environmental factors collectively shape the early‐life microbiome of calves, with Enterobacteriaceae emerging as the primary hosts of ARGs during this period. As calves mature, diet and age become the dominant regulators of the resistome and microbial function. Our findings revealed frequent interactions between MGEs and ARGs. Additionally, WT and ES exhibited a higher risk of ARG dissemination due to their elevated abundance and diversity of ARGs and MGEs. These insights advance our understanding of ARG transmission in livestock farming systems, providing a foundation for developing effective preventive and management strategies.

## METHODS

### Animals and study design

As shown in Figure 1A, a total of 136 Holstein dairy cows and 36 Holstein dairy calves were selected for this study. The farm trail was conducted at the Gansu Tianmu Farm (total animal head ≈ 18 thousands, Gansu Province, China) and Modern Dairy Farming (total animal head ≈ 19 thousands, Shaanxi Province, China). The feeding management and diet of cows (Table S1) were determined in accordance with the established norms of the tow farms. In Gansu, 100 healthy multiparous (second to fourth lactation) Holstein dairy cows with similar body conditions were selected for the study. Similarly, 36 healthy multiparous Holstein dairy cows with comparable body conditions were enrolled in Shaanxi. The offspring of these Shaanxi cows were also included in the study. Newborn calves in Shaanxi received CL from their dams within 1 h of birth. It is important to note that CL was not pasteurized to avoid affecting the structure and abundance of microbiome in CL. Then the calves were transferred to the clean and tidy separate calf hutches. Calves were fed milk from the age of 1–3 weeks, fed milk replacer (Table S2) from the age of 3–8 weeks, and weaned at 56 days of age. After weaning, the calves were maintained in calf hutches until the end of the experiment at 84 days of age. The calves had unrestricted access to water and starters (Table S3) from birth to 84 days of age.

### Collection of samples

As shown in Figure 1A, rumen fluid and fecal samples were collected from 136 postpartum cows within 30 min of calving in Gansu and Shaanxi provinces. Fresh CL was obtained from Shaanxi dairy cows within 1 h of calving using a milking machine. Fecal samples were also collected from the offspring of these Shaanxi cows at six time points (*n* = 126): at birth (ME, collected before CL administration), and on Days 1, 3, 35, 56, and 84 of age (collected before morning feeding). Rumen fluid samples were collected orally using a sterilized stomach tube and filtered through four layers of sterile gauze. Fecal samples were collected using sterile gloves. Environmental samples included WT samples and ES samples. The WT samples were collected four times (*n* = 4) from a cesspool near the calving pen during the experimental period and filtered through four layers of sterile gauze. The ES samples were obtained from five ground sampling sites near the calving pen in four times (*n* = 20), and the ES particles larger than 2 mm in diameter were further screened with a sterile sieve. All samples were stored in sterile tubes at –80°C for subsequent microbial analysis.

### DNA extraction, PCR amplification, and 16S rRNA‐amplicon sequencing

Microbial DNA was extracted from fecal samples using the DNeasy PowerLyzer PowerSoil Kit (Qiagen). DNA quality was assessed via NanoDrop 2000 spectrophotometry (Thermo Fisher Scientific) and 1% agarose gel electrophoresis. The V3‐V4 region of the bacterial 16S rRNA gene was amplified with primers 338 F (5′‐ACTCCTACGG GAGGCAGCAG‐3′) and 806 R (5′‐GGACTACHVGGGTWTCTAAT‐3′) using an ABI GeneAmp® 9700 thermocycler (Thermo Fisher Scientific). PCR products were gel‐purified with the AxyPrep DNA Gel Extraction Kit (Axygen Biosciences) and quantified using a Quantus™ Fluorometer (Promega Corporation). Paired‐end 250 bp sequencing was performed on an Illumina MiSeq PE250 platform (Illumina).

Raw FASTQ files were demultiplexed using an in‐house Perl script, quality‐filtered using fastp version 0.19.6, and merged using FLASH version 1.2.11 [64]. DADA2 was chosen to denoise the optimized sequences. Taxonomic assignment of amplicon sequence variants was then performed using the naive Bayes consensus taxonomy classifier implemented in Qiime2 and the SILVA 16S rRNA database (version 138) and adjusted for the rRNA operon copy number estimated using data from the rrnDB database [65].

### Metagenomic sequencing and processing

For library construction, DNA was fragmented to an average size of ~400 bp using a Covaris M220 ultrasonicator (Gene Company Limited). Subsequently, paired‐end libraries were generated with the NEXTFLEX Rapid DNA‐Seq kit (Bioo Scientific). Sequencing was performed on an Illumina NovaSeq. 6000 platform (Illumina) using a NovaSeq. 6000 S4 Reagent Kit, following the manufacturer's instructions. Raw sequencing reads underwent adapter trimming and removal of low‐quality reads (length < 50 bp; quality value < 20; or presence of N bases) using fastp (https://github.com/OpenGene/fastp, version 0.20.0) [64]. Reads were aligned to the *Bos taurus* genome, and any hits associated with the reads or their mated reads were removed. Quality‐filtered data were assembled using MEGAHIT (https://github.com/voutcn/megahit, version 1.1.2) [66]. Contigs with a length ≥ 300 bp were selected as the final assembling result. Open reading frames (ORFs) from each assembled contig were predicted using Prodigal (https://github.com/hyattpd/Prodigal, version2.6.3) [67], and ORFs ≥ 100 bp in length were retrieved. A nonredundant gene catalog with 90% sequence identity and 90% coverage was constructed using CD‐HIT (http://weizhongli-lab.org/cd-hit/, version 4.7) [68]. Gene abundance for each sample was estimated using SOAPaligner (https://github.com/ShujiaHuang/SOAPaligner, version.soap2.21release) with a 95% identity threshold [69]. Taxonomic classification of nonredundant genes was achieved by aligning them against the NCBI NR database using DIAMOND (http://ab.inf.uni-tuebingen.de/software/diamond/, version.2.0.11) [70] with an e‐value cutoff of 1e^–5^. Similarly, all filtered metagenomic reads were annotated against the Comprehensive Antibiotic Resistance Database (CARD, https://card.mcmaster.ca/) to identify ARGs. MGEs annotation for assembled gene bins was performed using Diamond (http://www.diamondserch.org/index.php,version0.8.35) against the MGEs90 database (https://bench.cs.vt.edu/ftp/data/databases/MGEs90.fasta) via BLASTP with an e‐value cutoff of 1e^–5^. For binning analysis, contigs exceeding 1000 bp were retained for subsequent binning with MetaBAT (https://bitbucket.org/berkeleylab/metabat, version 2.12.1), CONCOCT (https://github.com/BinPro/CONCOCT, version 0.5.0), and MaxBin2 (https://sourceforge.net/projects/maxbin/, version 2.2.5) to retrieve MAGs. Completeness, contamination, and strain heterogeneity of the bins were then assessed using CheckM (https://github.com/Ecogenomics/CheckM, version 1.0.12). ARGs colocalized on the same contig sequence within 5 kb of an MGE were considered potentially horizontally transferred.

### Metatranscriptomic sequencing and processing

Total RNA was extracted using the RNeasy PowerSoil Total RNA Kit (Qiagen). The samples were checked for degradation by 1% agarose gel electrophoresis, and the RNA concentration and purity were quantified using a NanoDrop 2000 (Thermo Fisher Scientific). Only samples with an RNA integrity number (RIN) > 7 were used to generate libraries. Total RNA was subjected to ribosomal RNA removal using the Ribo‐Zero rRNA Removal Kit (Illumina). The Illumina TruSeq Stranded mRNA LT Sample Prep Kit (Illumina) was used for RNA fragmentation, complementary DNA (cDNA) synthesis, cDNA library construction, polymerase chain reaction (PCR) amplification of DNA fragments with linkers, and library fragment selection and purification according to the manufacturer's instructions. Next, 1 μL cDNA libraries were used to be qualified by Agilent High Sensitivity DNA Kit using an Agilent Bioanalyzer 2100 (Agilent Technologies) and screened for linker‐free sequences with a single peak. The selected libraries were quantified with a Promega QuantiFluor RNA System (Promega Corporation) and further subjected to 2 × 150 bp double‐end sequencing when the content was above 2 nM. Paired‐end sequencing was performed with the barcoded libraries on the Illumina Novaseq. 6000 platform at Allwegene Technology using the NovaSeq. 6000 S4 Reagent Kit according to the manufacturer's instructions.

Raw sequencing reads first went through quality control using Trimmomatic [71], and clean reads were then aligned to the *Bos taurus* genome to exclude host and ribosomal RNA contamination. The screened mRNA sequences were assembled de novo and spliced using Trinity (version 2.5.1) and CD‐HIT software (version 4.7) to obtain unigenes [72]. The sequences were then aligned with the bacterial sequences in the NCBI nr database using BLASTX. MEGAN software (version 6.13.1) was used for species annotation, and QIIME (version1.7.0) was used to calculate the relative abundance at each classification level. The threshold value for gene identification was an e‐value < 1e^−5^.

### Statistical and bioinformatics analysis

Alpha diversity (Shannon and Chao1 indices) and bacterial abundance in each group were compared using the nonparametric Kruskal–Wallis test, followed by Dunn's multiple comparison test with Bonferroni correction for post‐hoc analysis. Beta diversity was assessed using Bray–Curtis dissimilarities and analyzed by analysis of similarity. LEfSe analysis was employed to identify signature ARGs from the high‐dimensional data. Significance thresholds of *p* < 0.05 and LDA score > 2 were used to define biomarkers. Data analysis was performed on the Majorbio Cloud platform (https://cloud.majorbio.com/) [73]. Visualization of diversity metrics was performed using the ggplot2 package (version 3.6.0) within the R environment. Additionally, density maps were created based on the Chao1 richness index of ARGs in rumen and feces for each cow across both regions. These maps were generated using an online analysis platform (https://www.genescloud.cn/home), with the x‐axis representing the richness range and the y‐axis depicting the number of cows corresponding to each richness level.

The NCM fits the observed relative abundance–frequency relationships in microbial resistome across various groups to beta distributions derived from neutral theory, thereby providing a robust framework for understanding community dynamics. The analysis was conducted using the packages of “Hmisc”, “minpack. lm” and “getopt” in “R” (version 4.2.3).

The principle of host identification of ARGs is as follows: Relative contribution of a taxon was represented by the relative abundance of a taxon participating in the resistome, which was calculated by summing the abundance of a taxon participating in the resistome assigned to that total abundance of all taxa involved in the resistome. Therefore, the sum of the abundances of all taxa/resistome detected in each subject was 1 [74].

Spearman's rank correlation coefficients between ARGs were calculated using the “psych” package within the R statistical environment (version 4.2.3). Only robust correlations with *p* < 0.05 and *R* > 0.7 were considered for constructing the ARG co‐occurrence network. The networks were visualized by Gephi (https://gephi.org/, version 0.9.2).

SourceTracker analysis was conducted within the R environment (version 4.2.3) to investigate the origin of microbiota in calf ME samples (sink). Maternal sources (rumen and feces) and environmental samples (WT and ES) were designated as potential sources. Employing a Bayesian framework, the analysis predicted the relative contribution of each source to the microbial community composition observed in the calf ME samples, based on the observed community structures within both source and sink samples.

The methodologies for assessing the potential ecological risks associated with the resistome were referenced from a prior study [25]. We defined the potential of ARGs to associate with MGEs and transfer to pathogens, as inferred from metagenomic data. A computational framework has been devised to evaluate each ARG, considering its relative abundance, mobility, and occurrence within a pathogen.

## AUTHOR CONTRIBUTIONS

**Shuai Liu**: Conceptualization; methodology; data curation; formal analysis; writing—original draft; writing—review and editing. **Yimin Zhuang**: Conceptualization; methodology; software; writing—original draft; writing—review and editing; formal analysis. **Tianyu Chen**: Conceptualization; methodology; data curation; writing—review and editing. **Duo Gao**: Methodology; data curation; formal analysis; writing—review and editing. **Jianxin Xiao**: Methodology; data curation; writing—review and editing; formal analysis. **Jinfeng Wang**: Conceptualization; writing—review and editing; visualization. **Jinghui Li**: Writing—review and editing; resources. **Xinjie Zhao**: Methodology; data curation; writing—review and editing. **Rong Peng**: Methodology; data curation; writing—review and editing. **Wenli Guo**: Methodology; data curation; Writing—review and editing. **Jialin Wei**: Methodology; data curation; writing—review and editing. **Mo Sha**: Methodology; data curation; writing—review and editing. **Jingjun Wang**: Data curation; writing—review and editing. **Jiaying Ma**: Data curation; writing—review and editing. **Mei Ma**: Project administration; resources; supervision. **Mengmeng Li**: Writing—review and editing. **Wei Wang**: Writing—review and editing. **Yajing Wang**: Writing—review and editing. **Shengli Li**: Writing—review and editing. **Zhijun Cao**: Conceptualization; methodology; validation; investigation; funding acquisition; writing—review and editing; project administration; supervision; resources.

## CONFLICT OF INTEREST STATEMENT

The authors declare no conflict of interest.

## ETHICS STATEMENT

The animal ethics application (No. AW42202702‐4‐1) was approved by the Animal Care and Use Committee of China Agricultural University.

## Supporting information


**Figure S1.** Fit of neutral model determined the contribution of deterministic and stochastic processes on ARGs in the four groups.
**Figure S2.** The correlation analysis.
**Figure S3.** CH index of enterotype robustness.
**Figure S4.** The Chao1 index and Shannon index of resistome among the three clusters.
**Figure S5.** The temporal dynamics of fecal microbiota in calves.
**Figure S6.** The age characteristics and driving factors of resistome.
**Figure S7.** Fit of neutral model determined the contribution of deterministic and stochastic processes on fecal microbiota at different ages.
**Figure S8.** The microbial diversity of colostrum, meconium, wastewater and soil.
**Figure S9.** The relative abundance of core MGEs and ARGs of the network among the four groups.
**Figure S10.** The potentially mobile analysis map of ARGs.
**Figure S11.** The potential ecological risks of resistome in the different niches.

**Table S1.** Nutritional components of cow feed.
**Table S2.** Nutritional components of milk replacer and milk.
**Table S3.** Ingredients and nutritional components of calf starter.

## Data Availability

The accession for the 16S sequencing and metagenomic data in this study are in the NCBI Sequence Read Archive (#PRJNA1078885, https://www.ncbi.nlm.nih.gov/bioproject/PRJNA1078885). and metatranscriptomic data is in the NCBI Sequence Read Archive (#PRJNA1052964, https://www.ncbi.nlm.nih.gov/bioproject/PRJNA1052964). The data and scripts used are saved in GitHub (https://github.com/z1164323345/imeta-data#). Supplementary materials (figures, tables, graphical abstract, slides, videos, Chinese translated version and update materials) may be found in the online DOI or iMeta Science http://www.imeta.science/.
