# Supplementary material for: Spatio‐temporal characteristics of the gastrointestinal resistome in a cow‐to‐calf model and its environmental dissemination in a dairy production system
Source: Imeta. 2025 May 14;4(4):e70047. doi: 10.1002/imt2.70047 (PMC12371271; doi:10.1002/imt2.70047)
Supplement: Supplementary file 1 — Figure S1. Fit of neutral model determined the contribution of deterministic and stochastic processes on ARGs in the four groups. Figure S2. The correlation analysis. Figure S3. CH index of enterotype robustness. Figure S4. The Chao1 index and Shannon index of resistome among the three clusters. Figure S5. The temporal dynamics of fecal microbiota in calves. Figure S6. The age characteristics and driving factors of resistome. Figure S7. Fit of neutral model determined the contribution of deterministic and stochastic processes on fecal microbiota at different ages. Figure S8. The microbial diversity of colostrum, meconium, wastewater and soil. Figure S9. The relative abundance of core MGEs and ARGs of the network among the four groups. Figure S10. The potentially mobile analysis map of ARGs. Figure S11. The potential ecological risks of resistome in the different niches. [file IMT2-4-e70047-s002.docx]

**Spatio-temporal characteristics of the gastrointestinal resistome in a cow-to-calf Model and its environmental dissemination in a dairy production system**

**Runing title:** Cow-calf gastrointestinal resistome spread in dairy systems

Shuai Liu ^1#^, Yimin Zhuang ^1#^, Tianyu Chen ^1#^, Duo Gao^1#^, Jianxin Xiao ^1,2#^, Jinfeng Wang ^3^, Jinghui Li ^4^, Xinjie Zhao ^1,5^, Rong Peng ^1,5^, Wenli Guo ^1^, Jialin Wei ^1,6^, Mo Sha ^1^, Jingjun Wang ^1^, Jiaying Ma ^1^, Mei Ma ^1^, Mengmeng Li ^1^, Wei Wang ^1^, Yajing Wang ^1^, Shengli Li ^1^, Zhijun Cao ^1*^

^1^ State Key Laboratory of Animal Nutrition and Feeding, International Calf and Heifer Organization, College of Animal Science and Technology, China Agricultural University, Beijing 100193, China.

^2^ Animal Nutrition Institute, Sichuan Agricultural University, Chengdu 611130, China

^3^ College of Food Science and Nutritional Engineering, China Agricultural University, Beijing 100083, China

^4^ Department of Medicine, Section of Genetic Medicine, University of Chicago, Chicago, IL 60637, USA

^5^ Institute of Agricultural Sciences, ETH Zurich, Zurich 8092, Switzerland

^6^ School of Biological Sciences, University of Bristol, Bristol BS81TQ, UK

^#^ These authors contributed equally: Shuai Liu, Yimin Zhuang, Tianyu Chen, Duo Gao, and Jianxin Xiao.

* Correspondence: [caozhijun@cau.edu.cn](mailto:caozhijun@cau.edu.cn) (Zhijun Cao)


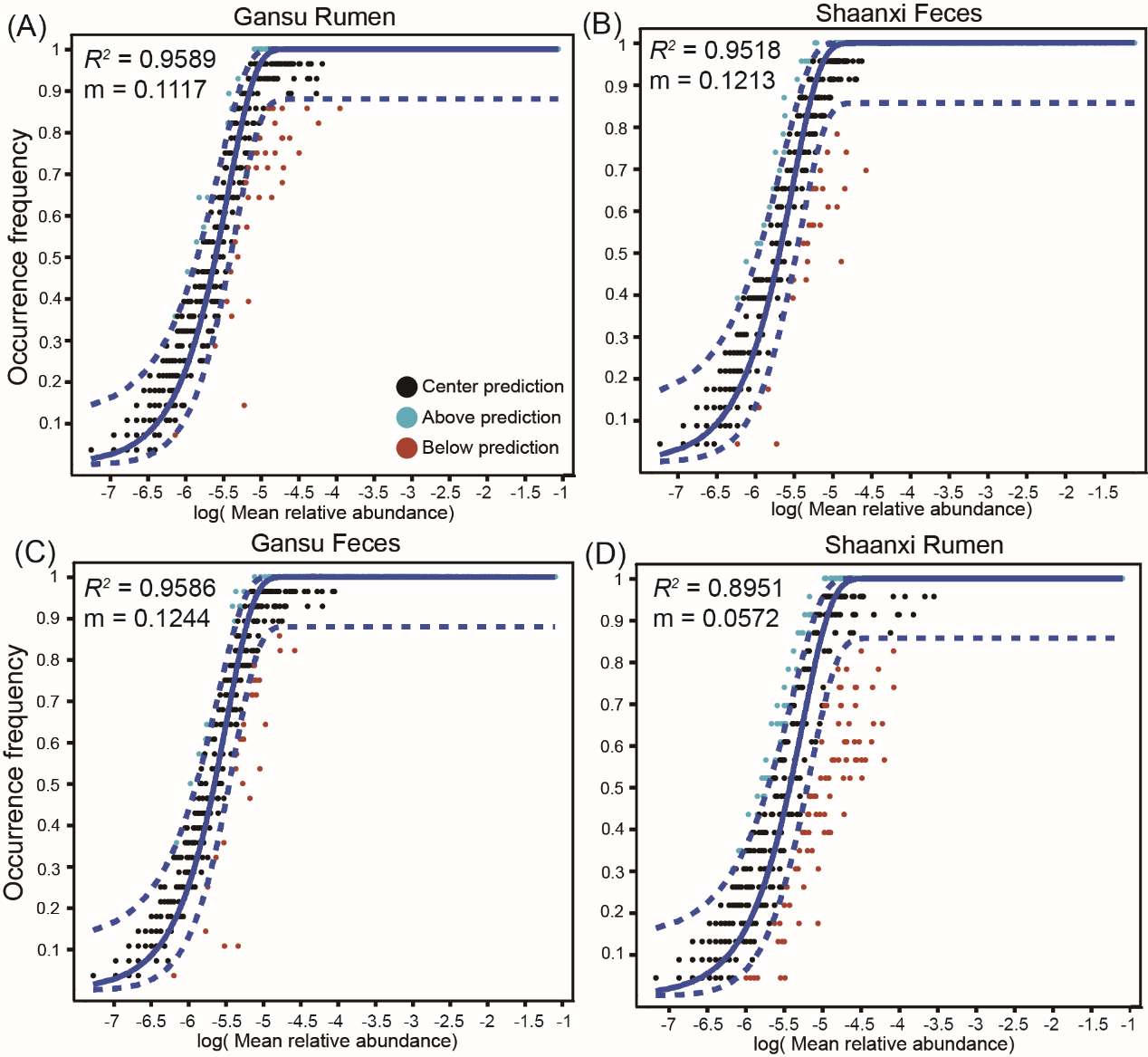


**Figure S1 Fit of neutral model determined the contribution of deterministic and stochastic processes on antibiotics resistance genes in the four groups.** (A) Rumen of calves in Gansu. (B) Feces of calves in Shaanxi. (C) Feces of calves in Gansu. (D) Rumen of calves in Shaanxi.


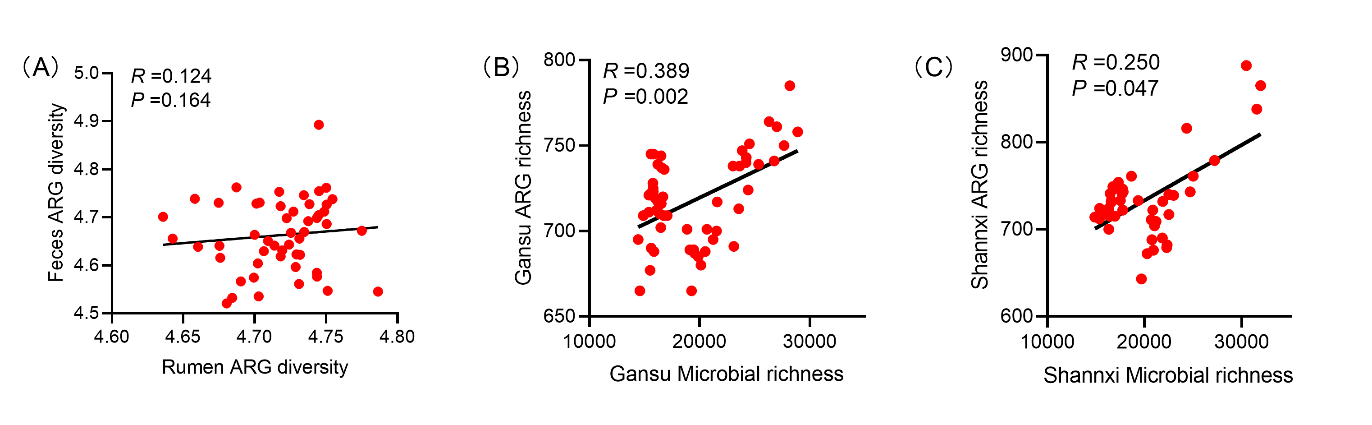


**Figure S2 The correlation analysis.** (A) feces antibiotics resistance genes (ARG) diversity vs rumen ARG diversity. (B) Gansu ARG richness vs Gansu microbial richness. (C) Shaanxi ARG richness vs Shaanxi microbial richness.


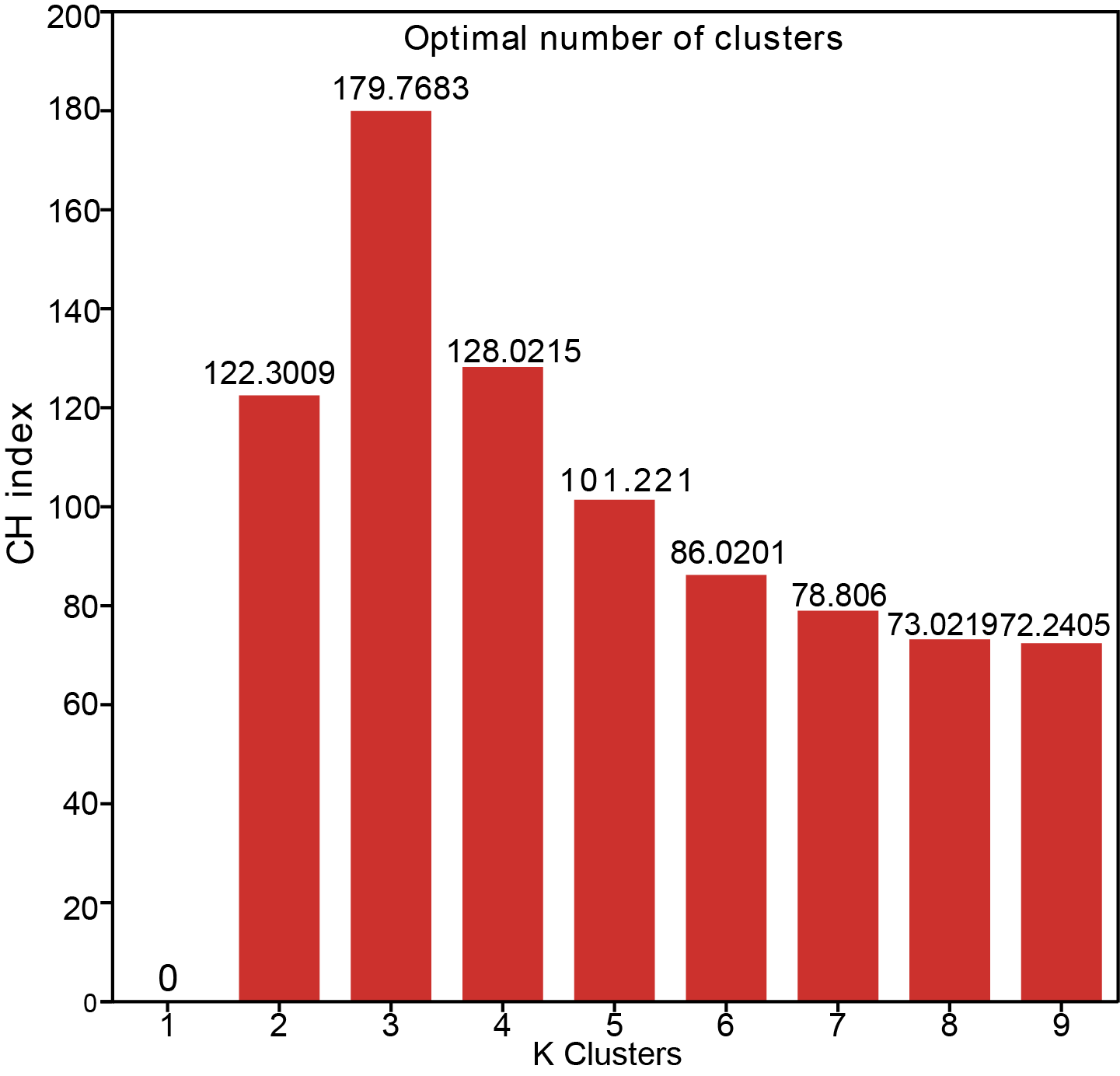


**Figure S3 CH index of enterotype robustness.** When the K clusters = 2, CH index showed the highest value indicating the accuracy of clustering division is the highest.


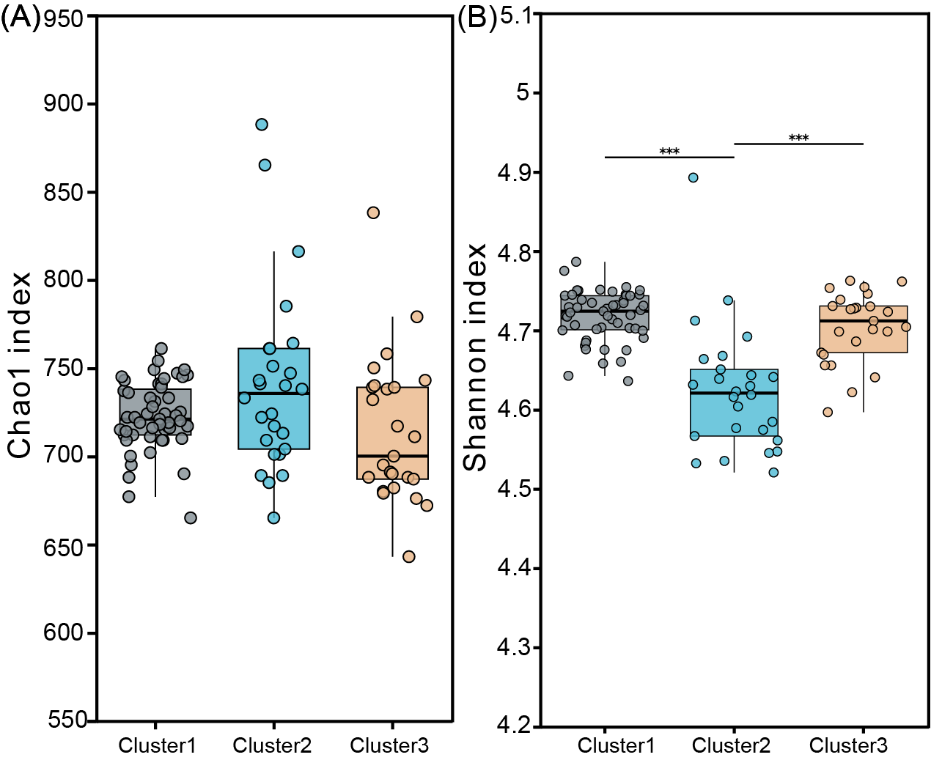


**Figure S4 The α diversity of resistome among the three clusters.** (A) The Chao1 index of the three groups. (B) The Shannon index of the three groups.


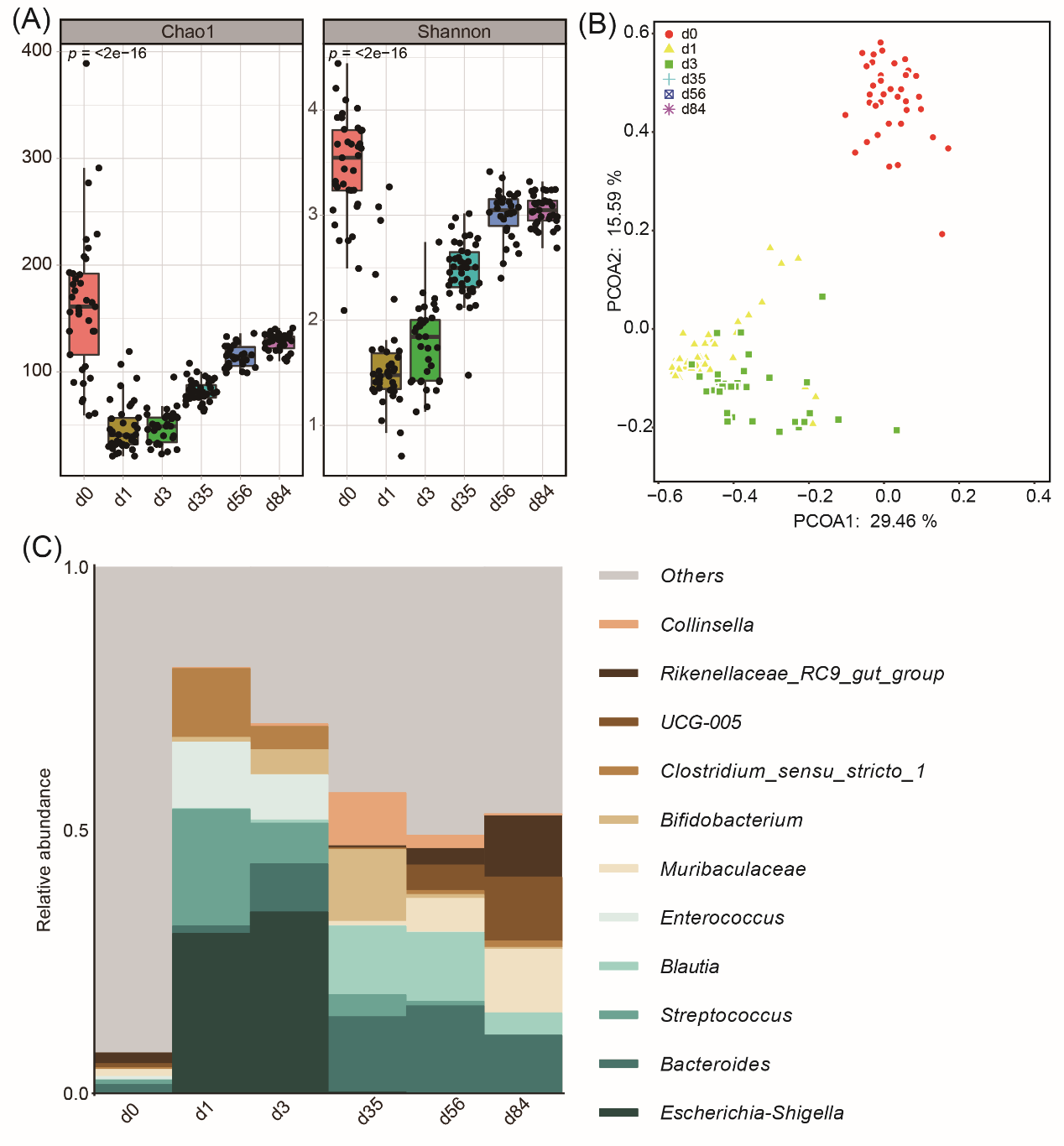


**Figure S5 The temporal dynamics of fecal microbiota in calves.** (A) The Chao1 index and Shannon index of fecal microbiota in calves at different timepoints. (B) PCoA based on the Bray- Curtis distance of microbiota in the feces of calves. (C) The microbial composition of fecal microbiota of calves at the genus level.


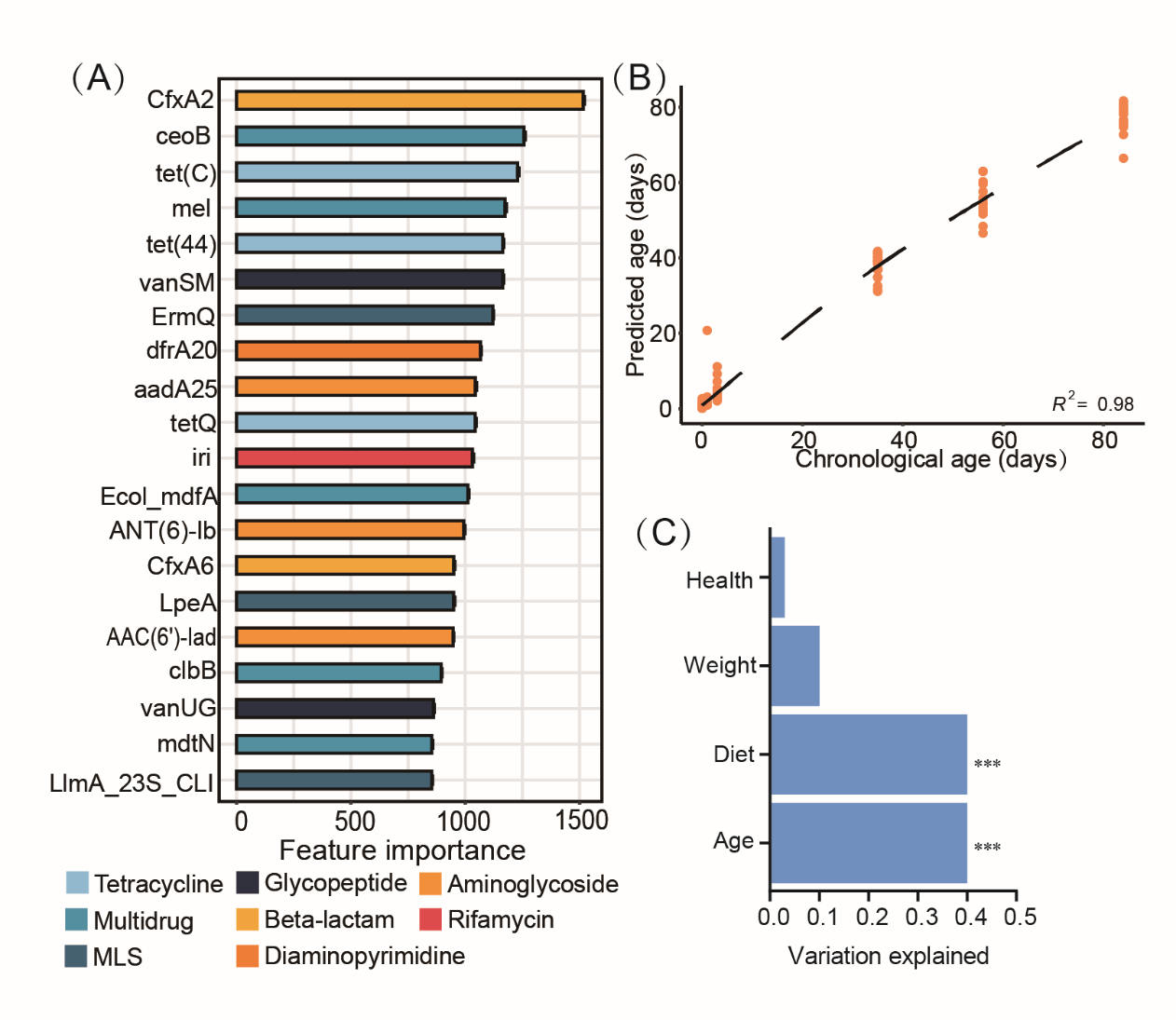


**Figure S6 The age characteristics and driving factors of resistome.** (A) The top 20 age-related antibiotics resistance genes (ARG) of the feces in calves were listed based on the mean square error of predictions (%IncMSE). (B) The distinct capability for age prediction from fecal ARGs of calves from 0 to 84 days of age. (C) Driving factors of temporal changes in calf resistome by Permutational multivariate analysis of variance (PERMANOVA).


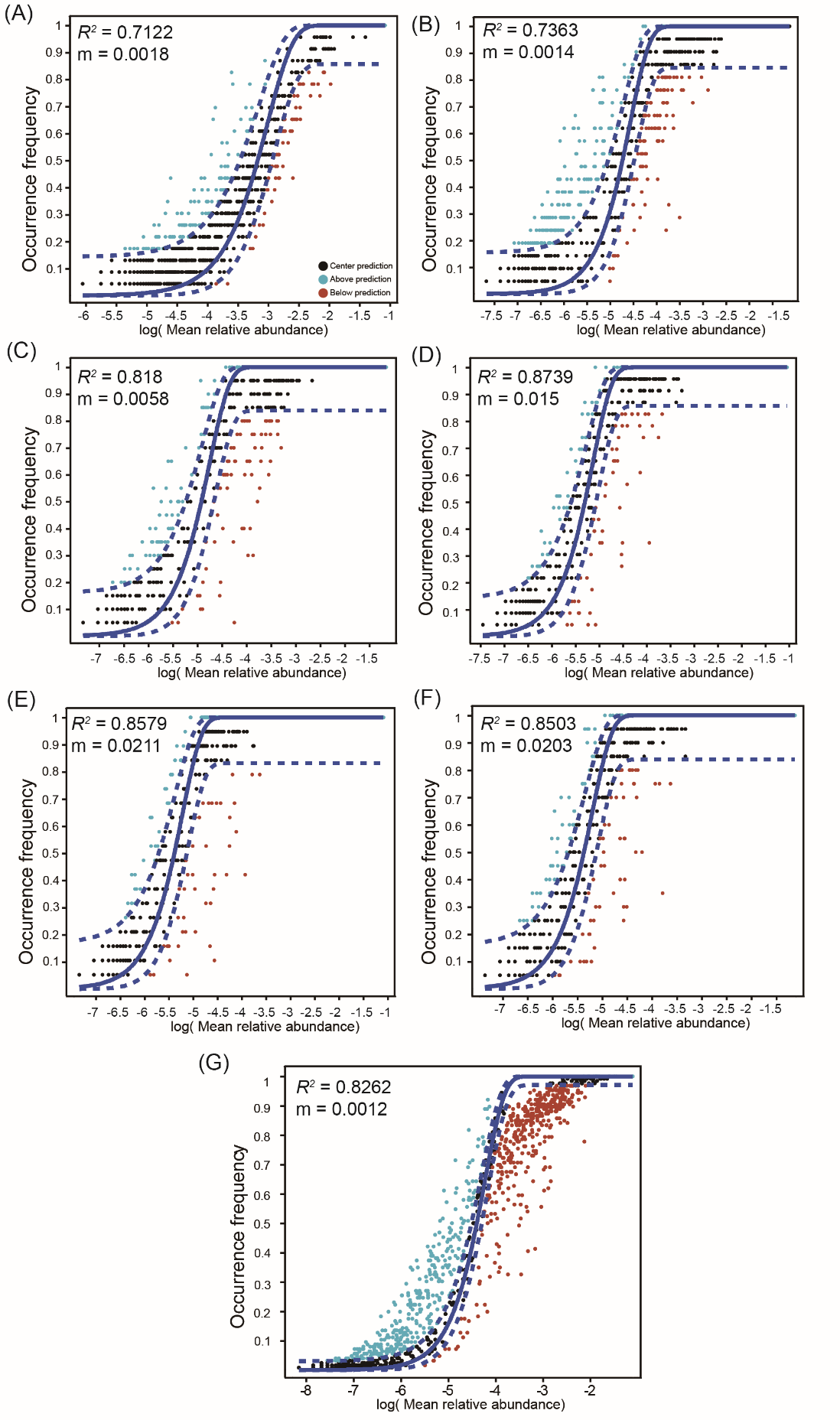


**Figure S7 Fit of neutral model determined the contribution of deterministic and stochastic processes on fecal microbiota at different ages.** (A) Calves at 0 d of age. (B) Calves at 1 d of age. (C) Calves at 3 d of age. (D) Calves at 35 d of age. (E) Calves at 56 d of age. (F) Calves at 84 d of age. (G) Calves across all the timepoints.


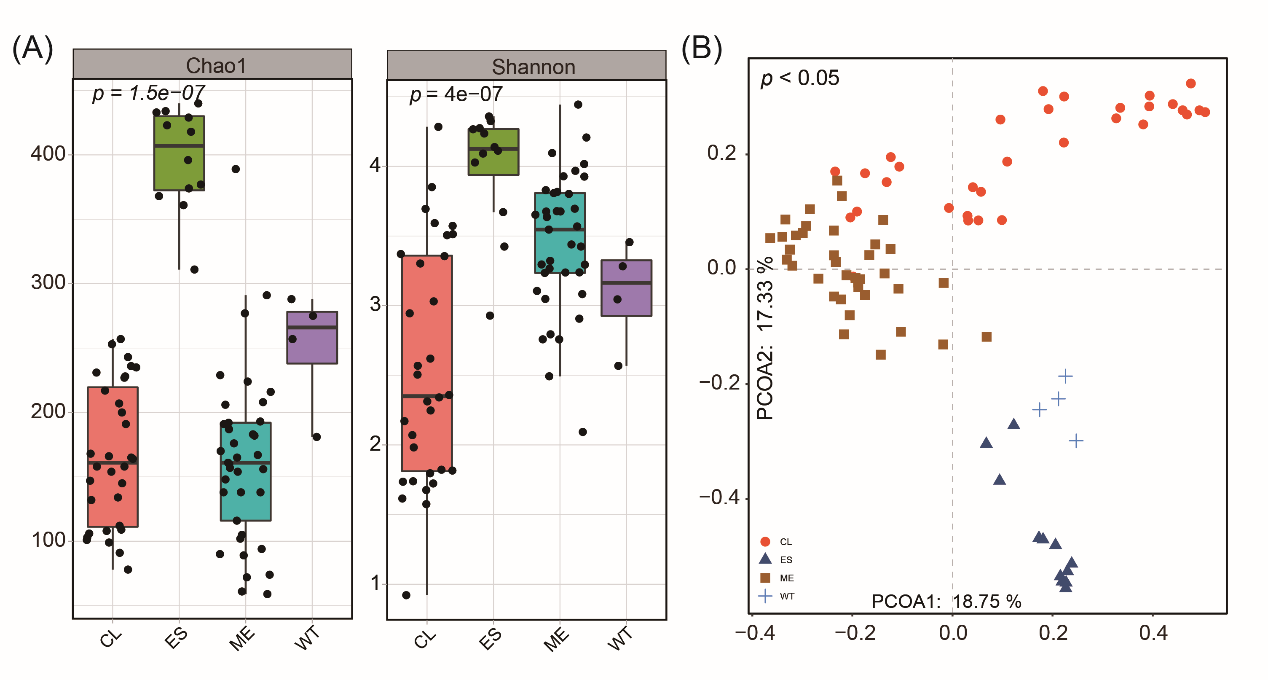


**Figure S8 The microbial diversity of colostrum, meconium, waste water and soil.** (A) The Chao1 index and Shannon index of microbiota in the four groups. (B) PCoA based on the Bray- Curtis distance of microbiota in the four groups.


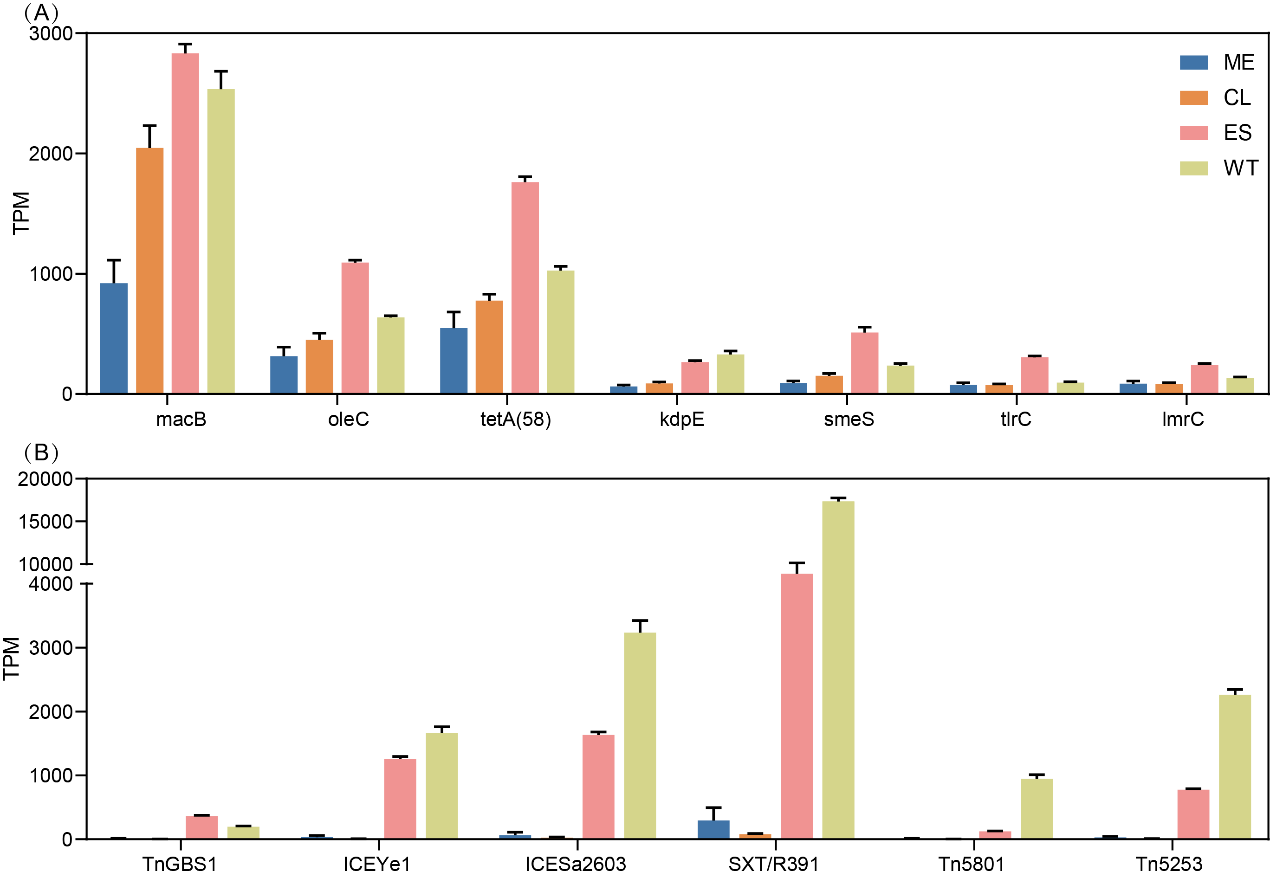


**Figure S9 The relative abundance of core MGEs and ARGs of the network among the four groups.** (A) The relative abundance of core MGEs. (B) The relative abundance of core ARGs. ARG = antibiotics resistance genes, MGE = Mobile genetic elements


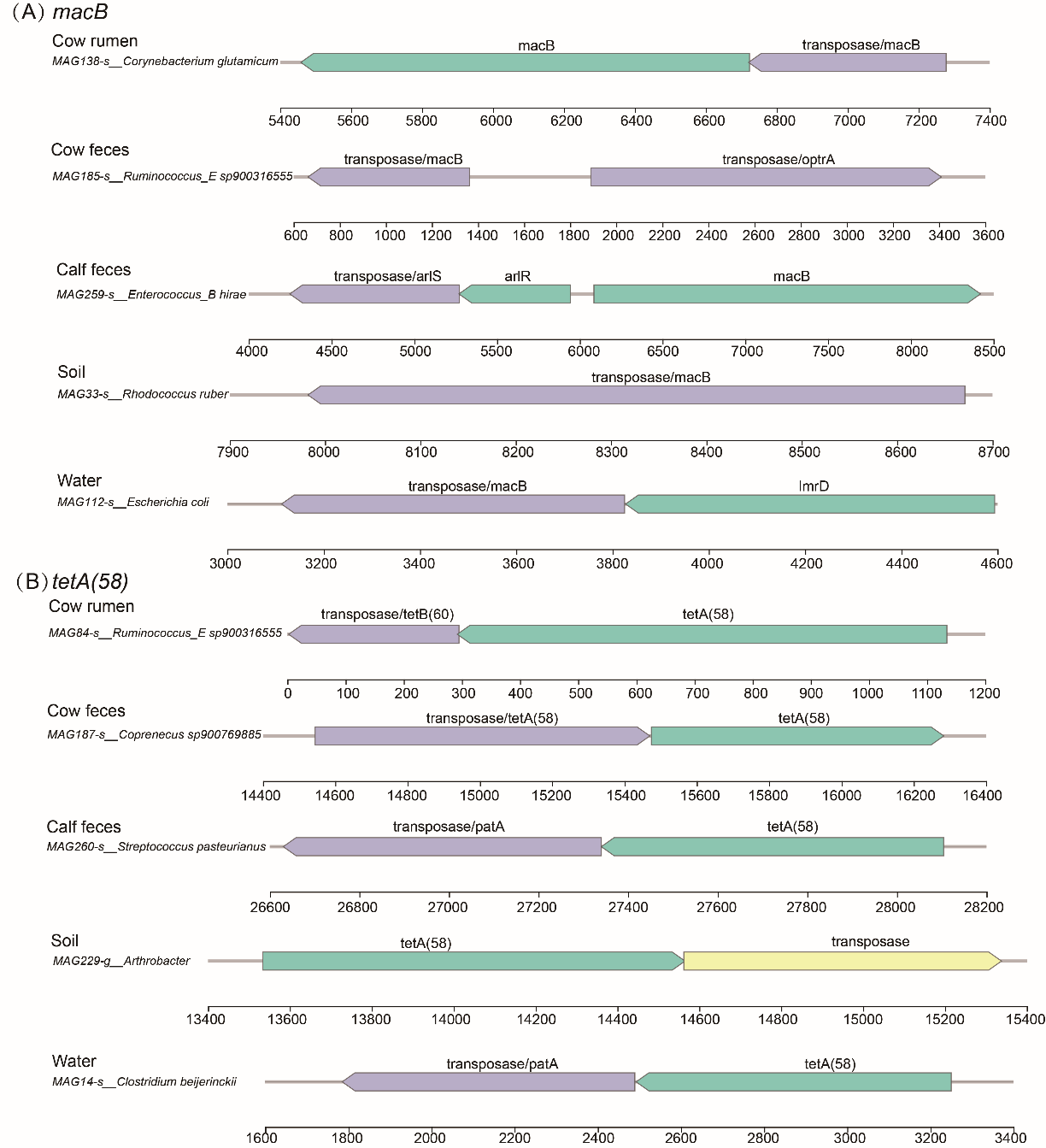


**Figure S10 The potentially mobile analysis map of ARGs.** (A) The potential horizontal gene transfer (HGT) prevalence of *macB* in the different niches. (B) The potential HGT prevalence of *tetA(58)* in the different niches.


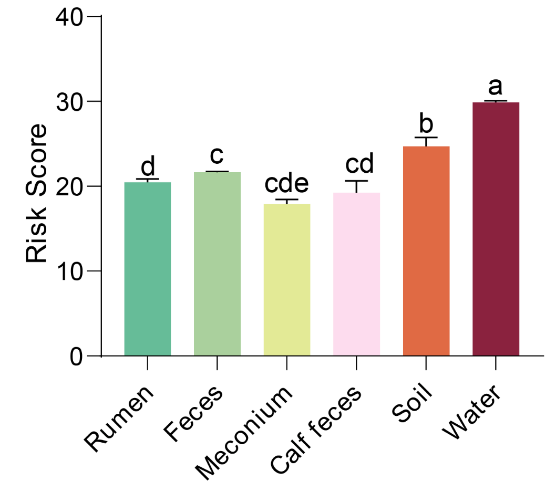


**Figure S11 The potential ecological risks of resistome in the different niches.** Different letters (a, b, c, d, e) on the histogram of each group indicate significant differences between groups (*p* < 0.05).
